# Association between PM_1_ Exposure and Lung Function in Children and Adolescents: A Systematic Review and Meta-Analysis

**DOI:** 10.3390/ijerph192315888

**Published:** 2022-11-29

**Authors:** Zhiqiang Zong, Mengjie Zhao, Mengyue Zhang, Kexin Xu, Yunquan Zhang, Xiujun Zhang, Chengyang Hu

**Affiliations:** 1 Department of Clinical Medicine, The Second School of Clinical Medicine, Anhui Medical University, Hefei 230032, China; 2 Department of Maternal, Child and Adolescent Health, School of Public Health, Anhui Medical University, Hefei 230032, China; 3 School of Public Health, Wuhan University of Science and Technology, Wuhan 430065, China; 4 Department of Epidemiology and Biostatistics, School of Public Health, Anhui Medical University, Hefei 230032, China; 5 Department of Humanistic Medicine, School of Humanistic Medicine, Anhui Medical University, Hefei 230032, China

**Keywords:** PM_1_, lung function, air pollution, cohort study, children, adolescents, meta-analysis

## Abstract

The detrimental effects of PM_2.5_ and PM_10_ (particulate matter less than 2.5 or 10 μm) on human respiratory system, including lung function, have been widely assessed. However, the associations between PM_1_ (particulate matter of less than 1 μm) and lung function in children and adolescents are less explored, and current evidence is inconsistent. We conducted a meta-analysis of the literature on the association between PM_1_ and lung function in children and adolescents to fill this gap. With no date or language constraints, we used a combination of MeSH (Medical Subject Headings) terms and free text to search PubMed, EMBASE and Web of Science databases through, 1 October 2022 for “PM_1_ exposure” and “lung function”. A total of 6420 relevant studies were identified through our initial search, and seven studies were included in our study. In this meta-analysis, the fixed effect and random effects statistical models were used to estimate the synthesized effects of the seven included studies. For every 10 μg/m^3^ increase in short-term PM_1_ exposure, forced vital capacity (FVC), forced expiratory volume in the first second (FEV_1_), peak expiratory flow (PEF) and maximal mid-expiratory flow (MMEF) decreased by 31.82 mL (95% CI: 20.18, 43.45), 32.28 mL (95% CI: 16.73, 48.91), 36.85 mL/s (95% CI: 15.33, 58.38) and 34.51 mL/s (95% CI: 19.61, 49.41), respectively. For each 10 μg/m^3^ increase in long-term PM_1_ exposure, FVC, FEV_1_, PEF and MMEF decreased by 102.34 mL (95% CI: 49.30, 155.38), 75.17 mL (95% CI: 39.61, 110.73), 119.01 mL/s (95% CI: 72.14, 165.88) and 44.94 mL/s (95% CI: 4.70, 85.18), respectively. Our study provides further scientific evidence for the harmful effects of PM_1_ exposure on lung function in children and adolescents, indicating that exposure to PM_1_ is detrimental to pulmonary health. To reduce the adverse health effects of air pollution on children and adolescents, effective preventive measures should be taken.

## 1. Introduction

It has been well documented that air pollution poses a great risk to human health. There were approximately 540 million people diagnosed with respiratory diseases worldwide in 2017, making it the third leading cause of death according to the Global Burden of Disease (GBD) 2020 report [1]. Air pollution in urban areas is primarily composed of particulate matter (PM), which poses a significant risk for respiratory diseases [2]. As compared with adults, children and adolescents are more susceptible to air pollution because their respiratory rates per body weight and lung surface area are greater [3,4]. Further, Ginsberg et al., 2005 demonstrated that the pulmonary region of the lung has a slower clearance rate, thus particles remain longer, resulting in a two to four-fold higher particle dose in children compared to adults [3]. In addition, due to their immature immune systems and developing lungs, children and adolescents are particularly susceptible to exposure to PM [5]. Several epidemiological studies have demonstrated that ambient PM exposure is associated with adverse health outcomes in children, including obesity, hypertension, metabolic syndrome, vision impairment, pneumonia, and decreased renal function [6,7,8,9,10,11].

The lung function of children and adolescents can be used to diagnose pulmonary diseases, which is an extremely important and measurable indicator of respiratory health [12,13]. The forced vital capacity (FVC) measures the volume of the lungs, while the forced expiratory volume in the first second (FEV_1_) measures the mechanical characteristics of large and medium airways [14]. When lung injury is in its early stages, alterations in FVC and FEV_1_ are usually observed. An analysis of peak expiratory flow (PEF) or maximum mid-expiratory flow (MMEF) may be useful in confirming small airway obstructions and monitoring diagnosis in cases in which other examinations are abnormal, such as asthma [15]. There is a general trend for PEF and MMEF to change when the lungs are affected by diseases.

Many previous studies have consistently reported that both short and long-term exposure to particle matters with aerodynamic diameter ≤ 2.5 μm (PM_2.5_) or particle matters with aerodynamic diameter ≤ 10 μm (PM_10_) were related to a decreased lung function [5,16,17,18,19,20,21,22]. Furthermore, particle matters with aerodynamic diameter ≤ 0.1 μm (PM_0.1_), also called ultrafine particles (UFPs), can result in systemic inflammation, endothelial dysfunction, and coagulation alterations that expose individuals to the risk for ischemic cardiovascular disease and hypertension [23]. Particulate matter with an aerodynamic diameter ≤ 1 μm (PM_1_) is a predominant component of PM. Evidence suggests that particles with a smaller size have a greater adverse health impact [8,9,11]. Despite this, relevant studies on the association of PM_1_ exposure with lung function parameters are inconsistent, and still lack sufficient evidence. In this regard, it is critical to evaluate the effects of higher PM_1_ levels on the respiratory systems of children and adolescents.

In order to identify surrogate markers of pulmonary health associated with PM_1_ exposure, we performed a systematic review and meta-analysis of studies that examined the relationship between PM_1_ exposure and metrics of lung function in children and adolescents.

## 2. Materials and Methods

This study was reported following the PRISMA (Preferred Reporting Items for Systematic Reviews and Meta-analyses) statement (Supplementary Materials, Table S1).

### 2.1. Study Question

Our study question was: “Among children and adolescents, how does a higher exposure to PM_1_ affect lung function compared with a lower exposure?”

### 2.2. Search Strategy

With the following keywords, we searched the PubMed, EMBASE, and Web of Science databases for eligible studies between the date of inception and 1 October 2022, that are representative of the exposure and outcome described in the following PECOS statement: (PM OR PM_1_ OR “particulate matter” OR “air pollution”) AND (“lung function” OR “pulmonary function” OR “respiratory system”). There is more detail in Supplementary Materials, Table S2 regarding the literature search terms used.

### 2.3. Study Selection

The study population (P) of interest consisted of children and adolescents between the ages of 6 and 18. The exposure of interest was PM_1_ (E), which was measured by an air pollution monitoring station or self-built environmental monitoring equipment. The exposure was expressed as for each unit increase (C). The indicators of lung function (O), including FVC, FEV_1_, PEF, and MMEF (FEF 25–75%), were used as outcomes, and studies should report the effect estimates (β coefficients) corresponding to PM_1_ exposure on these indicators. For inclusion, cross-sectional studies, cohort studies, case-control studies, and panel studies were considered. Only the most recent article was included in the case of duplicate publications.

**Figure 1:**
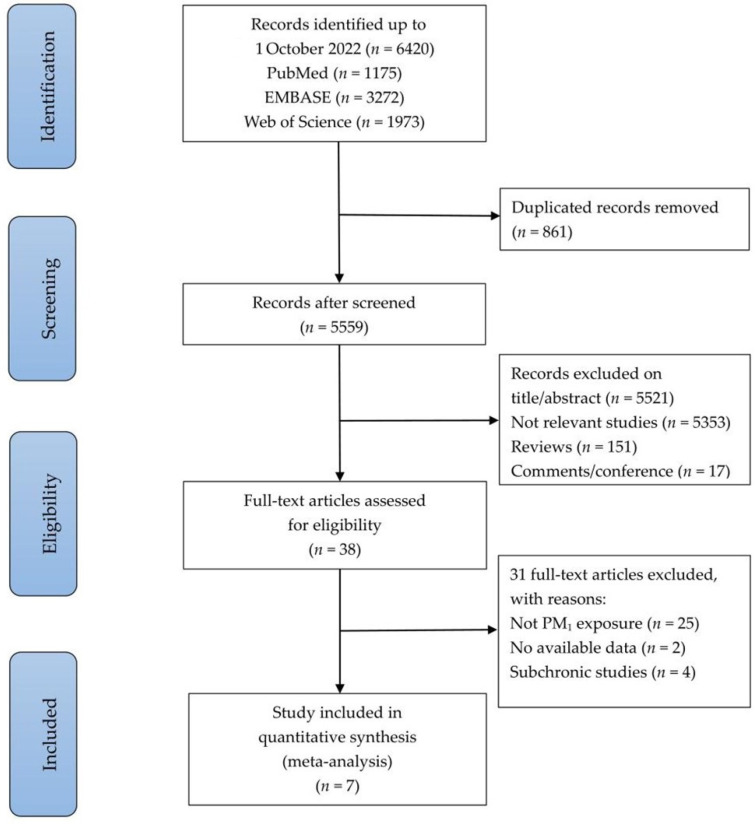
Flow chart of selection of studies included in the meta-analysis.

We considered only original studies for inclusion. Exclusion criteria were as follows: (1) neither effect estimates nor the ability to determine such estimates was provided; (2) research designs that involved intervention; and (3) sub chronic studies. Figure 1 illustrates the process of selecting studies. Each manuscript was initially screened based on its title and abstract, which was followed by an independent evaluation of its full text by two authors (Z.Z. and M.Z. (Mengyue Zhang)). In the event of disagreements, further discussions were held with the research team.

### 2.4. Data Extraction and Quality Assessment

We extracted information regarding the first author and publication year, study design, study location, sample size, meteorological data (temperature and relative humidity), PM_1_ exposure measurement, mean of PM_1_ concentration, statistical analysis model, exposure group, lung function indicators, adjusted covariates and main results. In order to conduct meta-analyses, we used the most fully adjusted effect estimates that represent the largest control for potential confounding factors. As necessary, authors were contacted directly to collect unpublished data.

A change in lung function indicators was reported for every 10 μg/m^3^ increase in PM_1_ concentration in our meta-analysis. The meta-analysis was conducted directly after incorporating the effect estimate if the study reported the change in lung function indicators per 10 μg/m^3^ increase. If the study reported the effect estimates for lung function indicators per inter quartile range (IQR) increase in PM_1_ exposure, the effect estimates were transformed as (10/IQR) *β and then entered into the meta-analyses. For the studies presented the percentage change (%) of lung function indicators for each 10 μg/m^3^ increase in PM_1_ concentration, we calculated the β value of lung function indicators by multiplying the mean lung function of all children or adolescents with the percentage (%). In previous studies, this method has also been used to convert data.

For the purpose of assessing the quality of studies included in the review, the Newcastle-Ottawa Scale (NOS) was used. An original version of this scale was developed to evaluate case-control and cohort studies. There is an adapted version that is widely used to evaluate cross-sectional studies [24]. In light of this review, we did not include case-control studies and we thus evaluated the three dimensions (selection, comparability and outcome) through different items for cohort and cross-sectional studies. Based on a NOS score of 0 to 9, studies with a score greater than 7 were considered to be of high quality, those with a score between 5 and 6 were considered to be of moderate quality, and any study with a score less than 5 was considered to be of low quality [25].

### 2.5. Statistical Analysis

Literature reports on four lung function indicators, including FVC, FEV_1_, PEF and MMEF (FEF 25–75%), as well as the exposure lengths of PM_1_ including long-term and short-term were recorded. Long-term exposure to ambient particulate matter is defined as exposure that lasts for more than three months. As described in a previous study, “medium-term exposure” refers to exposure to ambient particles for a period of 28 to 91 days [26]. “Short-term exposure” refers to exposure lasting less than 28 days to ambient particulate matter [26,27]. Unfortunately, we were unable to combine the results for lung function and medium-term exposure due to the limited data. To perform the meta-analysis, four lung function indicators and two duration periods of the exposure were used to categorize the exposures and outcomes into eight groups, named as changes in FVC/FEV_1_/PEF/MMEF due to short-term/long-term exposure to PM_1_.

Our study evaluated the pooled effect estimates for both long-term and short-term PM_1_ exposures using random effects and fixed effect models. The heterogeneity across study estimates was assessed using the I^2^ index, which was classified into low (25%), moderate (25–75%), and high (75%) categories [28,29]. In cases where heterogeneity among studies was greater than 50%, random effect models were used, while fixed effect models were used. To identify publication bias, Egger’s tests and funnel plots were used, and an Egger’s test *p*-value less than 0.05 was considered evidence of publication bias. In order to perform the sensitivity analysis, we deleted one study at a time from the pooled effect estimates. Generally, robust results can be defined as those that are similar to the primary results after excluding one study at a time from the meta-analysis. Stata version 15.1 (Stata Corp, College Station, TX, USA) was used for all analyses.

## 3. Results

### 3.1. Characteristics of Included Studies

According to the literature search, 6420 studies were identified, 861 duplicates were removed, and 5521 studies were excluded after titles and abstracts reviewed. The full-text evaluation of 31 studies resulted in their exclusion because (1) there were no available data on the effects of PM_1_ exposure; (2) a lack of adequate lung function indicators or exposure levels; (3) missing data on children or adolescents. Finally, a total of seven studies were included in the systematic review and meta-analysis (Figure 1) [30,31,32,33,34,35,36]. A total of three studies investigated the relationship between short-term PM_1_ exposure and lung function indicators, while four studies examined the relationship between long-term PM_1_ exposure and lung function indicators. Based on the quality assessment of seven studies, it was determined that all of them scored higher than 7 points, and were deemed to be of high quality (Table 1). More detailed quality assessment was presented in Supplementary Materials, Table S3.

For the three short-term exposure studies, one was cross-sectional study and two were panel studies. Of the three studies, one was conducted in East Asia (China) and the other two in Europe (Austria and Poland). The potential effects of climate parameters have only been considered in one study, and lag effects have only been considered in two studies. There was a wide age range for the study population in the eligible studies, ranging from 9 to 18 years of age. The mean PM_1_ concentrations in the included studies ranged from 15.3 to 47.4 μg/m^3^. In the long-term exposure studies, four were cross-sectional studies conducted in China, and only one of them took climate parameters into account. Among the participants in the long-term exposure studies, the age range was between 7 and 14 years. The average PM_1_ concentrations in the long-term exposure studies ranged from 46.8 to 47.5 μg/m^3^.

In this meta-analysis, four lung function indicators—FVC, FEV_1_, PEF, and MMEF—were included, and lung function data were obtained from the measurement of spirometers. The data on short-term exposure to PM_1_ were obtained through fixed site instrument measurement, whereas long-term exposure data were obtained primarily from ground monitoring stations. Confounding factors such as sex, age, body mass index (BMI), parental education, household income and physical activity were adjusted in each included study. In both short-term and long-term exposure studies, recent respiratory infections, indoor coal use for cooking or heating and indoor tobacco smoke exposure have generally been adjusted.

### 3.2. Primary Meta-Analysis

**Figure 2:**
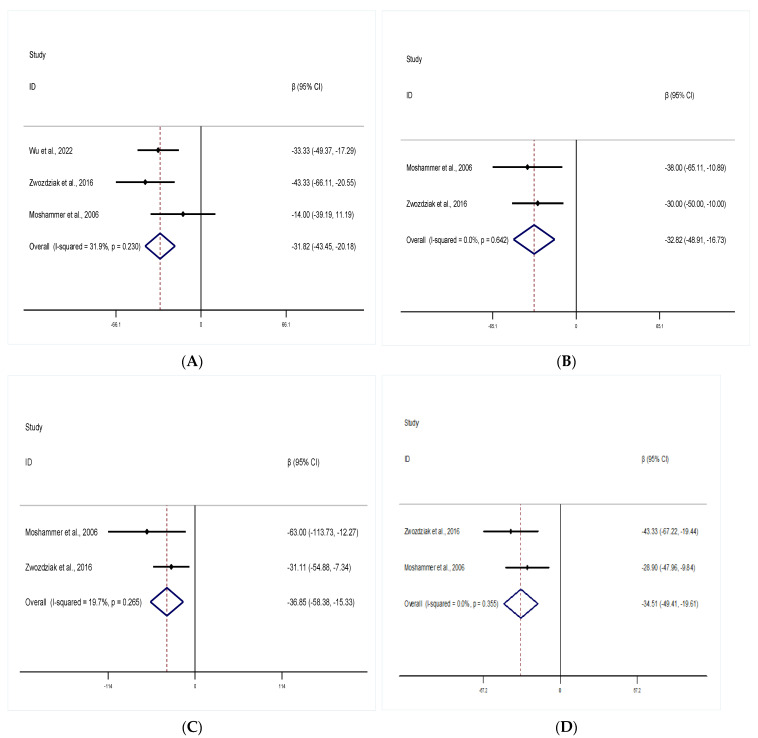
Forest plot of the meta-analysis: per 10 μg/m^3^ increase of PM_1_ was associated with pooled β values of lung function indicators in the short-term group: (**A**) FVC, (**B**) FEV_1_, (**C**) PEF, (**D**) MMEF [31,32,36]. The open diamonds represent the combined β value for each group. The solid line represents β value = 0.

A total of three studies assessed the association of PM_1_ and indicators of lung function in the short-term exposure group. Per 10 μg/m^3^ increase in PM_1_ exposure was associated with a decrease in indicators of lung function. Meta-analytical effect estimates on three studies assessed FVC was as follows: −31.82 mL (95% CI: −43.45, −20.18; I^2^ = 31.9%) with fixed effect model was applied (Figure 2A). Meta-analytical effect estimates on two studies assessed FEV_1_ was as follows: −32.82 mL (95% CI: −48.91, −16.73; I^2^ = 0.0%) with fixed effect model was applied (Figure 2B). Meta-analytical effect estimates on 2 studies assessed PEF was as follows: −36.85 mL/s (95% CI: −58.38, −15.33; I^2^ = 19.7%) with fixed effect model was adopted (Figure 2C). Meta-analytical effect estimates on two studies assessed MMEF was as follows: −34.51 mL/s (95% CI: −49.41, −19.61; I^2^ = 0.0%) with fixed effect model was used (Figure 2D).

**Figure 3:**
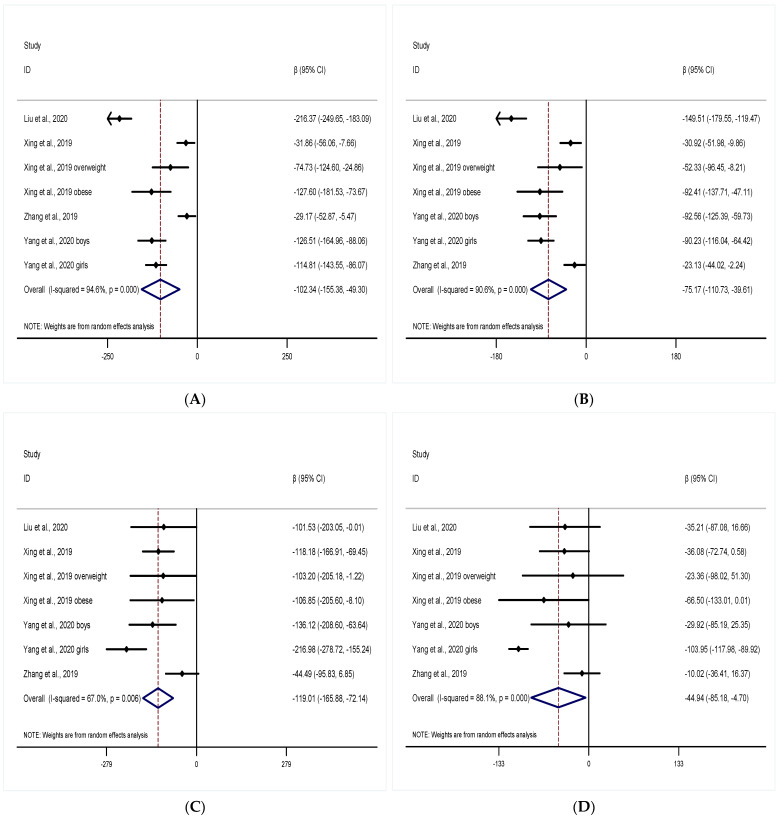
Forest plot of the meta-analysis: per 10 μg/m^3^ increase of PM_1_ was associated with pooled β values of lung function indicators in the long-term group: (**A**) FVC, (**B**) FEV_1_, (**C**) PEF, (**D**) MMEF [30,33,34,35]. The open diamonds represent the combined β value for each group. The solid line represents β value = 0.

A total of four studies evaluated the association of PM_1_ and indicators of lung function in the long-term exposure group. Per 10 μg/m^3^ increase in PM1 was associated with a decrease in indicators of lung function. Meta-analytical effect estimates on four studies evaluated FVC was as follows: −102.34 mL (95% CI: −155.38, −49.30; I^2^ = 94.6%) with random effects model was applied (Figure 3A). Meta-analytical effect estimates on four studies assessed FEV_1_ was as follows: −75.17 mL (95% CI: −110.73, −39.61; I^2^ = 90.6%) with random effects model was applied (Figure 3B). Meta-analytical effect estimates on four studies assessed PEF was as follows: −119.01 mL/s (95% CI: −165.88, −72.14; I^2^ = 67.0%) with random effects model was adopted (Figure 3C). Meta-analytical effect estimates on four studies evaluated MMEF was as follows: −44.94 mL/s (95% CI: −85.18, −4.70; I^2^ = 88.1%) with random effects model was used (Figure 3D).

### 3.3. Publication Bias

**Figure 4:**
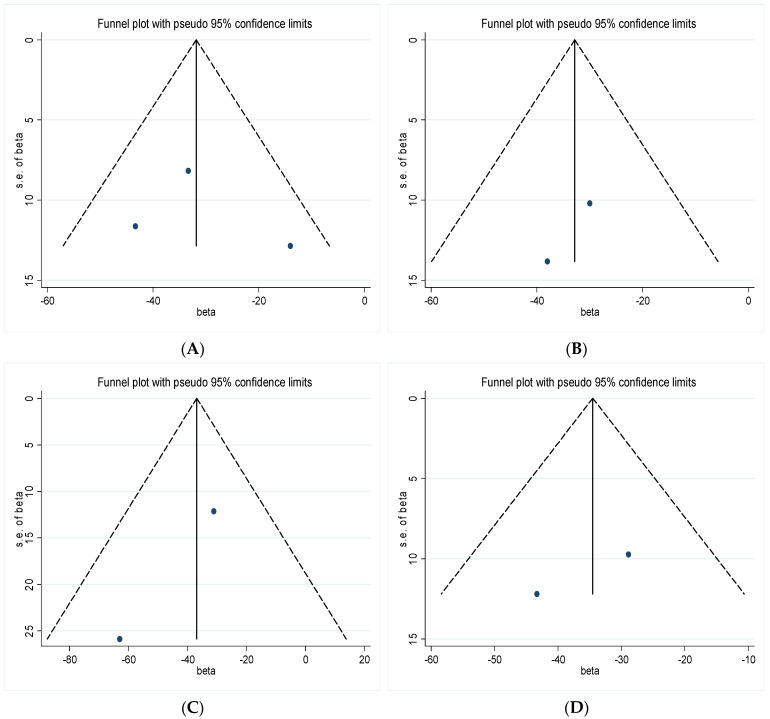
Funnel plot of the effects of PM_1_ on lung function indicators in the short-term exposure group: (**A**) FVC, (**B**) FEV_1_, (**C**) PEF, (**D**) MMEF.

**Figure 5:**
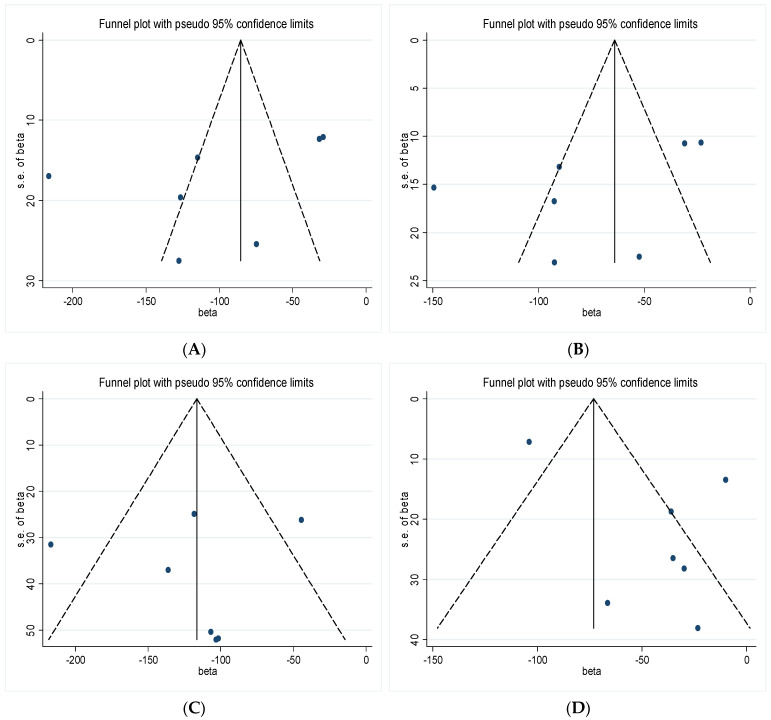
Funnel plot of the effects of PM_1_ on lung function indicators in the long-term exposure group: (**A**) FVC, (**B**) FEV_1_, (**C**) PEF, (**D**) MMEF.

In both short-term and long-term exposure groups, vertical funnel plots and Egger’s tests were used to analyze publication bias for PM_1_ and lung function indicators. Visually, all funnel plots were essentially symmetrical (Figure 4 and Figure 5). In short-term exposure studies, the *p* value of Egger’s test was 0.767 for FVC, while Egger’s tests were not available for FEV_1_, PEF and MMEF due to the small number of studies included. In long-term exposure studies, the *p* values of Egger’s tests were 0.247 for FVC, 0.221 for FEV_1_, 0.826 for PEF, and 0.107 for MMEF, respectively, which indicated that there was no evidence of publication bias for these analyses.

### 3.4. Sensitivity Analysis

**Figure 6:**
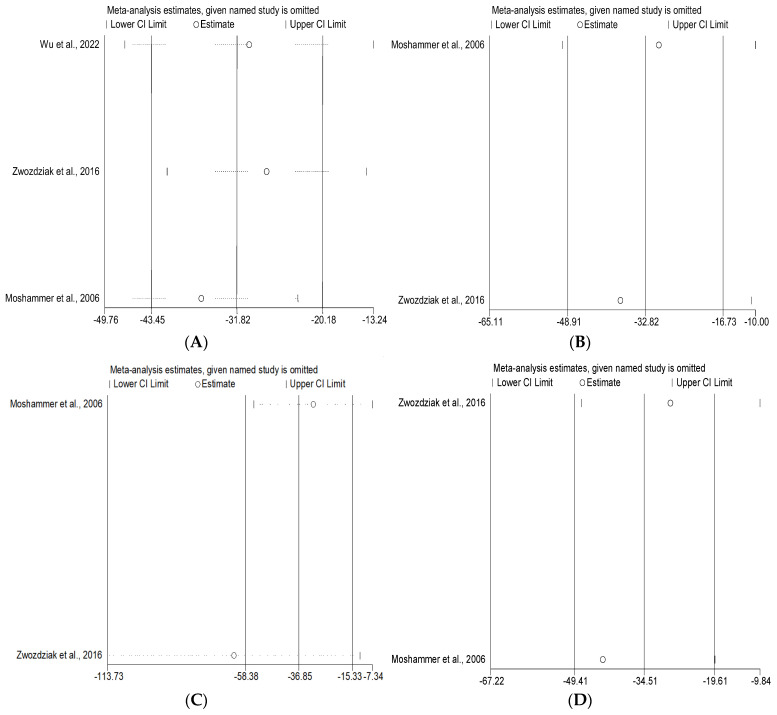
Sensitivity analyses of included studies in the short-term exposure group: (**A**) FVC, (**B**) FEV_1_, (**C**) PEF, (**D**) MMEF [31,32,36].

**Figure 7:**
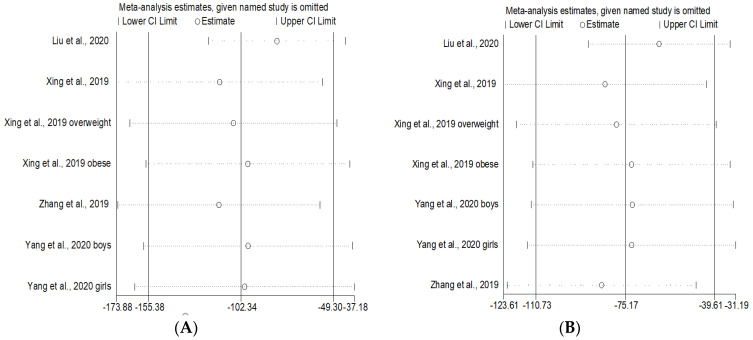

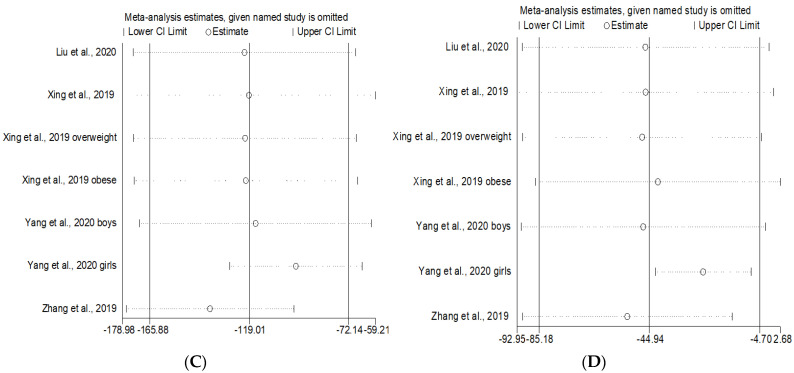
Sensitivity analyses of included studies in the long-term exposure group: (**A**) FVC, (**B**) FEV_1_, (**C**) PEF, (**D**) MMEF [30,33,34,35].

We performed the sensitivity analyses by using the leave-one-out method to assess the stability of the results. In short-term PM_1_ exposure studies, results of the sensitivity analyses indicated that, with the exception of PEF group, the pooled effect estimates were not significantly affected by excluding each individual study (Figure 6). In long-term PM_1_ exposure studies, results of the sensitivity analyses showed all pooled effect estimates were robust (Figure 7).

## 4. Discussion

To our knowledge, this is the first meta-analysis that examines the relationship between PM_1_ exposure and lung function in children and adolescents. Observations indicate that both short-term and long-term exposure to PM_1_ results in decreases in four lung function indicators (FVC, FEV_1_, PEF, and MMEF), which are commonly used to measure obstructive and restrictive lung disease. Exposure over a long period of time has a more pronounced effect on lung function compared to short-term exposure.

Due to the fact that PM_1_ and PM_2.5_ are the components of PM_2.5_ and PM_10_ respectively, it is possible that PM_1_ has posed great threat to human health in comparison with similar changes of other components of PM_2.5_ and PM_10_ [8,37,38,39]. Several previous epidemiological studies have shown that PM_1_ has larger effect on lung function compared to the same changes in PM_2.5_ and PM_10_ [22,30,31,33,34,35]. The adverse effects of PM_1_ exposure on pulmonary health may be explained by several biological mechanisms, but they are not entirely understood. As finer aerosol atmospheric particles, the diameter of PM_1_ is much smaller than PM_2.5_ and PM_10_, which enables it to reach deeper part of lungs, and has a larger surface area-to-volume ratio, as well as a higher level of adsorbent or condensed toxic compounds per unit mass [40]. Therefore, it is more appropriate to use mass as a measure of PM_1_ rather than particle numbers. The smaller the PM fraction, the greater the potential for detrimental biological interactions with the lungs [35]. Acute exposure to PM can cause inflammation of the lungs [41,42,43]. A longitudinal panel study in the pediatric population revealed the short-term impact of PM_1_ on lung function, which was attributed to elevated fractional exhaled nitric oxide, an inflammatory biomarker of airway [22,44,45]. Additionally, toxicological evidence has demonstrated that PM_1_ particles are more detrimental to health than PM_2.5_ particles in terms of cytotoxicity and inflammation [46]. Apart from causing inflammation in the alveolus and alveolar ducts, inhalation of PM can cause endothelial dysfunction and enhance the production of oxidative stress, resulting in pulmonary dysfunction [47].

In the meta-analysis, significant heterogeneity was detected in some of the analyzed PM_1_ and lung function indicator combinations. It is possible that differences in gender, BMI (normal weight, overweight, and obese), influenza vaccination, and breastfeeding may contribute to the observed heterogeneity. As one of the factors potentially influencing lung function, gender plays an important role [48]. Yang et al. reported that girls had significantly lower lung function levels than boys following long-term exposure to PM_1_, and that they were more susceptible to it [34]. Various factors may influence gender differences in lung function in response to air pollution, including differences in the development of the lungs and airways, with males having larger lungs and a larger number and area of alveoli at birth [48]. Previous research has shown that higher estrogen production in adolescence increases the risk of lung disease in girls [49]. It has been found that PM_1_ exposure and obesity have significant interactions with respiratory symptoms and asthma in children [33]. Increased adipose tissue can produce adipokines, and adipocytes can release cytokines that cause inflammation and oxidative stress, which, in turn, cause the deterioration of lungs [50,51,52,53,54]. The presence of PM in the blood can also lower insulin sensitivity, promote the dysfunction of β-cells, and stimulate the production of adipokines [55,56,57]. As reported by Liu et al., influenza vaccination may have the potential to moderate the adverse effects of ambient PM_1_ exposure on lung function of children [30]. Vaccination against influenza may modify associations in children through mechanisms that are not well understood. There is a possibility that influenza viruses can amplify the negative effects air pollutants have on lung function, resulting in more severe respiratory problems. As a result, influenza vaccination may reduce the risk of co-exposure to influenza and air pollution, thereby providing protection for children [58,59]. The findings of Zhang et al. indicate that breastfeeding may reduce the incidence of lung function impairments among Chinese children exposed to air pollution, including PM_1_ [35]. Compared to bottle feeding or early solid food consumption, breast milk may promote greater pulmonary immune growth and maturation due to its high content of immunological components, including cytokines, chemokines, antibodies derived from the mother, and leukocytes [60,61,62]. One of the most significant benefits of breastfeeding may be that it will postpone the onset of lung infections and diseases, as breast milk contains anti-infective components that can only boost passive immunity and may not provide protection once breastfeeding has ended [35].

Our study had several limitations that need to be noted. It is important to note that only a very small number of effect estimates were obtained from the included studies on each lung function indicator, as the association between PM_1_ and lung function has only recently attracted interest. However, when two effect estimates were included in a meta-analysis, it was considered acceptable [63], and there was a large number of systematic reviews and meta-analyses of high quality have been published in the field of environmental health, especially the first to investigate a specific exposure-outcome association [64,65,66]. Second, the relatively small number of studies included in the analysis precludes us from conducting further subgroup analyses based on the influence of various factors. We only consider the four lung function indicators FEV_1_, FVC, PEF, and MMEF. It is not possible to evaluate the association between PM_1_ and lung function with other lung function indicators, such as FEF25%, FEF75%, or FEV_1_/FVC, due to the lack of relevant data. Furthermore, we were unable to explore the lag effects of PM_1_ exposure on lung function indicators when considering the short-term exposure pattern. A similar assessment of the lag years of long-term exposure to PM_1_ on lung function was also not possible. Third, in the present study, we focused only on the value in PM_1_ itself and it was now well aware of the dominant role of aerosol numbers (ultrafine particle number concentration, condensation nuclei larger than 10 nm, etc.) over mass for PM_1_. However, due to the lack of routinely monitored relevant data, few studies have considered these issues and further studies are encouraged to explore the associations between health outcomes and other particle metrics rather than particle mass. Last but not least, this meta-analysis only evaluated studies that were conducted in China and Europe, and we can note that the exposure levels of mean PM_1_ concentrations (ranging from 15.3 to 47.4 μg/m^3^ in short-term, 46.8 to 47.5 μg/m^3^ in long-term) were relatively low, which limited the ability to generalize the results to other countries or regions with severe air pollution.

## 5. Conclusions

Our findings indicate that both short-term and long-term exposure to PM_1_ is associated with impaired lung function in children and adolescents, while long-term exposure has a more profound effect than short-term exposure. Our findings contribute to the scientific evidence for the harmful effects of short- and long-term PM_1_ exposure on the respiratory health of children and adolescents. Therefore, appropriate protective measures must be taken to mitigate the detrimental effects of air pollution on human health, particularly for the susceptible populations.

## Figures and Tables

**Table 1:** Basic characteristics of the studies included in the meta-analysis.

First Author and Publication Year	Study Design	Country	Sample Population	Meteorological Data	PM_1_ Exposure Measurement	Mean of PM_1_ Concentration	Statistical Analysis Model	Exposure Group	Lung Function Indicators	Adjusted Covariates	NOS Score
Liu et al., 2020 [30]	Cross-sectional study	China	6740 children aged 7–14 years	Temperature: 8.4 °C Relative humidity: 62.0%	Monitoring by a monitoring station	46.8 μg/m^3^	Linear regression	Long-term exposure	FVC, FEV_1_, PEF, MMEF	Age, gender, parental education, household income, environmental tobacco smoke exposure, BMI category, annual average temperature and annual average relative humidity	8
Moshammer et al., 2006 [31]	Panel study	Austria	163 children aged 7–10 years	Temperature: − Relative humidity: −	Monitoring by a monitoring station	15.03 μg/m^3^	Generalized Estimating Equations model	Short -term exposure	FVC, FEV_1_, PEF, MMEF	Sex, age, height and weight	7
Wu et al., 2022 [32]	Cross-sectional study	China	35,334 students aged 9 to 18 years	Temperature: − Relative humidity: −	Fixed site instrument monitoring	47.4 μg/m^3^	Distributed lag non-linear models	Short-term exposure	FVC	Gender, age, body mass index (BMI) category, residence, month of the survey, intake of eggs, intake of milk, physical activity, and screen time	7
Xing et al., 2019 [33]	Cross-sectional study	China	4518 children with normal weight, 1068 with overweight, 1154 with obese	Temperature: − Relative humidity: −	Monitoring by a monitoring station	47.5 μg/m^3^	Linear regression model	Long-term exposure	FVC, FEV_1_, PEF, MMEF	Age, gender, smoking exposure, parental education, breastfeeding status, income, home coal use, house pet, family history of atopy, temperature during investigation, and study district	8
Yang et al., 2020 [34]	Cross-sectional study	China	6740 children aged 7–14 years	Temperature: − Relative humidity: −	Monitoring by a monitoring station	47.5 μg/m^3^	Linear regression model	Long-term exposure	FVC, FEV_1_, PEF, MMEF	Age, body mass index, breast fed status, gender, parental education, income, passive tobacco smoke exposure, home coal use, house pet, house renovation, and family atopy	8
Zhang et al., 2019 [35]	Cross-sectional study	China	1989 children with not breastfed, aged 7–14 years	Temperature: − Relative humidity: −	Monitoring by a monitoring station	46.8 μg/m^3^	Linear regression model	Long-term exposure	FVC, FEV_1_, PEF, MMEF	Age, sex, height, birth weight, preterm birth, parental education, annual family income, exercise per week, passive smoke exposure, home coal use, presence of a house pet, home renovation in the past 2 years, area of residence per person, asthma diagnosis, family history of atopy, and short-term air pollution concentrations	8
Zwozdziak et al., 2016 [36]	Panel study	Poland	141 school children aged 13–14 years	Temperature: 18−21 °C Relative humidity: 31−54%	Fixed site instrument monitoring	22.0 μg/m^3^	Generalized estimating equations model	Short-term exposure	FVC, FEV_1_, PEF, MMEF	Sex, smoking, dampness, street, dust, pollen, mold, traffic	7

## Data Availability

The data presented in this study are available on request from the corresponding authors.

## Supplementary Materials

The following supporting information can be downloaded at: https://www.mdpi.com/article/10.3390/ijerph192315888/s1, Table S1: Preferred Reporting Items for Systematic reviews and Meta-Analysis (PRISMA) 2009 Checklist; Table S2: Database search term list; Table S3: Quality evaluation using Modified Newcastle Ottawa Scale (NOS) for 7 observational studies included in the meta-analysis.
